# Transparent Memory For Harsh Electronics

**DOI:** 10.1038/srep44429

**Published:** 2017-03-14

**Authors:** C. H. Ho, J. R. Durán Retamal, P. K. Yang, C. P. Lee, M. L. Tsai, C. F. Kang, Jr-Hau He

**Affiliations:** 1 Department of Electrical and Computer Engineering, Purdue University, West Lafayette, Indiana 47907, USA; 2 Computer, Electrical and Mathematical Sciences and Engineering (CEMSE) Division, King Abdullah University of Science & Technology (KAUST), Thuwal 23955-6900, Saudi Arabia

## Abstract

As a new class of non-volatile memory, resistive random access memory (RRAM) offers not only superior electronic characteristics, but also advanced functionalities, such as transparency and radiation hardness. However, the environmental tolerance of RRAM is material-dependent, and therefore the materials used must be chosen carefully in order to avoid instabilities and performance degradation caused by the detrimental effects arising from environmental gases and ionizing radiation. In this work, we demonstrate that AlN-based RRAM displays excellent performance and environmental stability, with no significant degradation to the resistance ratio over a 100-cycle endurance test. Moreover, transparent RRAM (TRRAM) based on AlN also performs reliably under four different harsh environmental conditions and 2 MeV proton irradiation fluences, ranging from 10^11^ to 10^15^ cm^−2^. These findings not only provide a guideline for TRRAM design, but also demonstrate the promising applicability of AlN TRRAM for future transparent harsh electronics.

“Harsh electronics” have received more attention recently due to the increasing demands by nuclear, medical, aerospace, and military applications that require devices which can operate in extreme environments of ionizing radiation^1^. Electronics that are regularly called upon to withstand such conditions include sensors^2^, transistors^3^, and memories^4^. However, long-term exposure of these devices under high energy proton and ion irradiation can cause the device to degrade, resulting in catastrophic failure of the entire electronic system^5^. For example, high proton irradiation fluences on photodetectors reduce the photo-to-dark current ratio down to zero due to the enhanced dark current^6^,^7^. Strong ionizing doses on high-electron-mobility transistors shift the threshold voltage and generate large leakage currents due to hole trapping in the barrier and gate dielectric layers, respectively^8^. Finally, the effect of highly energetic particle radiation on floating-gate transistors, the leading non-volatile memory (NVM) technology, can cause single-event upsets, burst errors, and stuck bits, which can be attributed to the high sensitivity of the charge storage mechanism to radiation-induced defects and charging^9^. Among these applications, NVM devices have received the most attention because they are indispensable for any electronic system. To make them more durable, researchers have developed a new type of NVM – resistive random access memory (RRAM) – which offers an alternative storage mechanism based on the resistive switching properties of electrically-triggered conductive nano-filaments (CNFs). RRAM is unique among memory technology in that it offers robust radiation-tolerant operation^1^,^10^.

Furthermore, RRAM is considered the most promising candidate for next generation NVM not only due to its simple device structure, high density, fast switching, low power, long-term retention, and endurance, but also because of its multiple advanced functionalities, such as transparency, flexibility, stretchability, wearability, and environmental friendliness^11^. Transparent RRAM (TRRAM) has particularly stood out owing to its key role in building “invisible circuits” by integrating functional wide-band gap materials^12^,^13^, thus enabling see-through data storage devices for display and industrial applications.

Despite these remarkable features of TRRAM, its severe performance degradation under harsh environments remains a critical issue. For example, the ambient environment, including interfacial chemisorption, moisture, and atmospheric corrosion, induces measurable changes in the device’s operation voltage, resistance states, and reliability^14^,^15^,^16^,^17^. To mitigate these environmental instabilities, researchers have developed several strategies, including the use of surface passivation layers^18^ and optimized doping profiles^14^.

However, these methodologies have only focused on materials surface engineering. To provide long-term device reliability of RRAM and TRRAM devices, it would be more efficient to use materials that are already stable in high radiation conditions. To this end, we chose to study aluminum nitride (AlN) due to its direct bandgap (6.2 eV)^19^, high thermal conductivity (134 W/cm K) and thermal stability^20^, high breakdown field (0.95 MV/cm)^21^, and superior radiation hardness^22^,^23^,^24^, making it an ideal material for harsh electronics. Previously, researchers demonstrated an AlN-based photodetector that was stable at high temperature and in radiative environments, verifying the potential of these AlN-based devices in extreme conditions^6^. Moreover, since transparent conducting indium tin oxide (ITO) is highly resistant to proton damage for fluences up to 10^16^ ions cm^−2 ^25, ITO/AlN/ITO TRRAM has also been reported to exhibit nanosecond switching and low-power operation^12^. To build on these developments, it is important to understand and explicitly evaluate the switching characteristics of TRRAM made with such materials under extreme environmental conditions for use in harsh electronics.

In this study, we investigated the reliability of ITO/AlN/ITO TRRAM under various conditions, including different environments (vacuum, air, N_2_, and O_2_) and proton irradiation fluences. Our results indicate that the device features a high-to-low resistance ratio (*R*_*H*_*/R*_*L*_) of 30 without significant degradation after 100 endurance cycles, validating its excellent durability and resistive switching capability even during such extreme exposures. Furthermore, statistical analyses, including cycle-to-cycle and device-to-device tests of 50 cells, verify the excellent switching stability and uniformity under high oxygen partial pressure and proton irradiation. These experimental results demonstrate the potential of AlN TRRAM for applications in harsh electronics due to the device’s superior reliability in extreme environments.

## Results and Discussion

**Figure 1:**
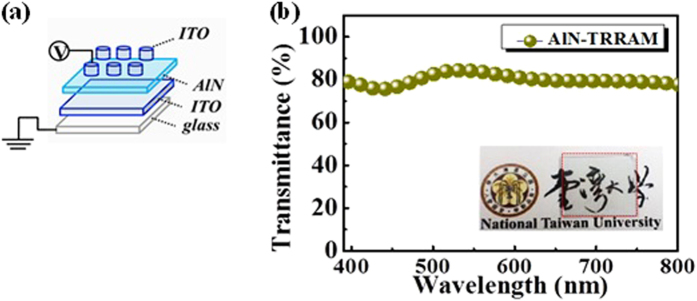
(**a**) The ITO/AlN/ITO sandwich configuration of the two-terminal TRRAM device. **(b)** The transmittance spectrum of the ITO/AlN/ITO structure within the visible region (400 nm to 800 nm). The inset shows a photograph of the as-fabricated device marked with a red rectangle. The background depicts the National Taiwan University logo, which can be observed through the device without any refraction or distortion.

To quantitatively examine transparency, we investigated the transmittance spectrum of the as-fabricated TRRAM device, which was composed of alternating layers of indium tin oxide (ITO) and AlN on a glass substrate (*i.e.*, ITO/AlN/ITO/glass). As shown in Fig. 1b, the average transmittance of the device was 80% within the visible wavelength region (400 nm to 800 nm). In the inset of Fig. 1a, we demonstrate the transparent nature of the device (marked by a red rectangle), beneath which the National Taiwan University logo can be clearly observed.

**Figure 2:**
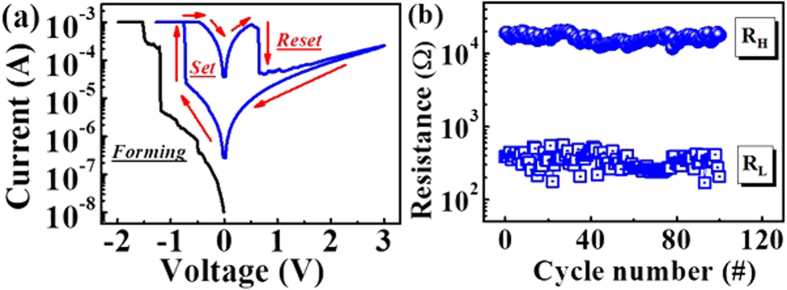
(**a**) Typical *I-V* characteristics of an AlN TRRAM device under atmospheric conditions. **(b)** The corresponding DC endurance of the device over 100 cycles.

Figure 2a shows the bipolar resistive switching characteristics of AlN-based TRRAM. During the measurements, we applied a DC voltage to the top electrode while the bottom electrode was grounded. The current compliance was set to 1 mA to prevent permanent destruction of the dielectric thin films during the forming process, in which the resistance of the device transitioned from an initial resistance state to a low resistance state for the first time. As shown in Fig. 2a, the current jump at −1.5 V indicates the completion of the forming process. By sweeping the voltage above a positive threshold value (*V*_*RESET*_ = 0.8 V). a sudden decrease in the current was then observed (as denoted by the Reset arrow in Fig. 2a), indicating that the device has been switched to a high resistance state (*R*_*H*_). When we subsequently decreased the voltage below a negative threshold value (*V*_*SET*_ = −0.8 V), there was an abrupt jump in the current (as denoted by the Set arrow in Fig. 2a), which indicated that the device had been switched to a low resistance state (*R*_*L*_). We then performed a 100-cycle endurance test to extract the statistical distributions of the resistance states, *R*_*H*_ and *R*_*L,*_ and the operation switching voltages (*V*_*SET*_ and *V*_*RESET*_), respectively. Each cycle was composed of the set and reset processes, as indicated in Fig. 2a, interleaved with voltage pulses of 0.1 V/100 ms to read the current after each set and reset process. Figure 2b demonstrates the endurance properties of AlN TRRAM, in which the resistance of both *R*_*H*_ and *R*_*L*_ showed only small fluctuations over 100 cycles. These results demonstrate the reversible and steady bipolar resistive switching (RS) characteristics of AlN TRRAM.

This bipolar switching characteristics can be explained according to the metal nitride-based RS mechanism, which relies on the formation/rupture of CNFs *via* nitride-related electron trapping/detrapping processes^12^,^26^. At the forming and set processes, an electro-reductive/electromechanical process triggered by the forward electric field causes the electrons to be trapped at crystal defects, leading to the creation of nitrogen vacancies through interstitial nitrogen ion release^27^. Nitrogen ions then migrate/drift toward the metal-nitride/electrode interface while the nitrogen vacancies, located at ~0.25 eV below the conduction band of AlN^27^, trap injected electrons, enabling trap-to-trap electron hopping through the CNFs. As a result the device enters the *R*_*L*_. Conversely, at the reset process the local Joule heating effect and reverse field cause electron detrapping events from nitrogen vacancies, leading to the annihilation of nitrogen vacancies through nitrogen fixation, and thus blocking trap-to-trap electron hopping at the ruptured area. As a result the device enters *R*_*H*_.

**Figure 3:**
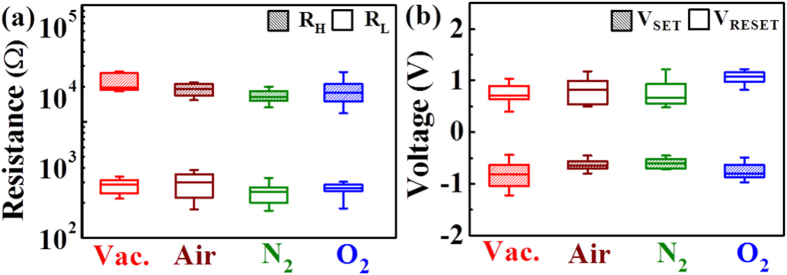
The **(a)** *R*_*H*_ and *R*_*L*_ and **(b)** *V*_*SET*_ and *V*_*RESET*_ distributions of the AlN TRRAM devices under ambient conditions of vacuum (Vac.), air, nitrogen (N_2_), and oxygen (O_2_).

Furthermore, it is well-known that the chemisorption of oxygen acts as an electron trap for charge carriers on the surface of metal oxides, which leads to an increase in the surface potential and deterioration of the device’s performance. To examine the surface effects of AlN TRRAM, we performed a 100-cycle endurance test of the device under four ambient conditions to simulate nitrogen-rich (air and N_2_) and nitrogen-poor (vacuum and O_2_) environments. We conducted a statistical analysis of 50 cells in order to evaluate the high and low resistance states (*R*_*H*_ and *R*_*L*_) and switching voltage distributions (*V*_*SET*_ and *V*_*RESET*_), as shown in Fig. 3a and b, respectively. Although the resistance in both states and switching voltages remained fairly stable with a high-to-low resistance ratio (*R*_*H*_/*R*_*L*_) around one order of magnitude under all ambient conditions, we observe that nitrogen-rich and -poor environments had reverse effects on the variability of the operating voltages (*ΔV*_*SET*_*and ΔV*_*RESET*_) and resistances states (*ΔR*_*L*_*and ΔR*_*H*_). Under a nitrogen-rich environment, *ΔR*_*H*_ and *ΔV*_*SET*_were reduced, while *ΔR*_*L*_ and *ΔV*_*RESET*_ increased. According to the previously explained RS mechanism of AlN, it is reasonable to assume that the nitrogen-rich environment exerts a high nitrogen partial pressure into the CNFs. As a consequence, nitrogen vacancies are steadily annihilated during the reset process, which results in nearly uniform rupture of the CNFs during subsequent reset cycles. Such stabilization reduces the fluctuations of *R*_*H*_ and anchors *V*_*SET*_, leading to the reduced values of *ΔR*_*H*_ and *ΔV*_*SET*_, respectively. In contrast, the high nitrogen partial pressure alters the transient currents in the *R*_*L*_ due to undesired nitrogen vacancy annihilation events that compromise the stability of the CNFs and randomize *ΔV*_*RESET*_, leading to our observation of the increased *ΔR*_*L*_ and *ΔV*_*RESET*_values.

On the other hand, under a nitrogen-poor environment, we observe that *ΔR*_*L*_ and *ΔV*_*RESET*_ became lower, while *ΔR*_*H*_ and *ΔV*_*SET*_ increased. To explain this opposite effect, we recall not only the nitride-related RS mechanism of AlN, but also the tunable electrical characteristics of ITO *via* the modulation of the oxygen concentration^28^. For example, oxygen-deficient polycrystalline ITO can act simultaneously as a reservoir of oxygen vacancy sites and oxygen ions, and hence the CNFs could be extended into the ITO electrode during the forming and set processes^29^,^30^,^31^. In fact, the oxygen deficient properties of ITO allow the injection/extraction of oxygen ions at the AlN/ITO interface owing to the high solubility of oxygen in the AlN lattice^32^, which could result in the formation/dissolution of an AlO_x_ barrier interlayer. Indeed, according to the high chemical affinity of oxygen towards aluminum, it is likely that a thin layer of alumina is present on the surface of the AlN samples^23^. Therefore, the high oxygen content under the nitrogen-poor environment constrains further dissolution of the AlO_x_ barrier interlayer during the set processes, leading to fluctuating transient currents at the high resistance state that increase the *V*_*SET*_ mean value and *ΔR*_*H*_^33^. Conversely, the high oxygen content stabilizes the reformation of the AlO_x_ barrier interlayer during the reset process, leading to the reduced *ΔR*_*L*_. Despite the small differences in variability under nitrogen-rich or -poor environments, the environmentally stable results shown in Fig. 3 clearly indicate that the detrimental surface effects on the RS characteristics of metal oxide-based RRAM can be suppressed with the use of AlN films.

**Figure 4:**
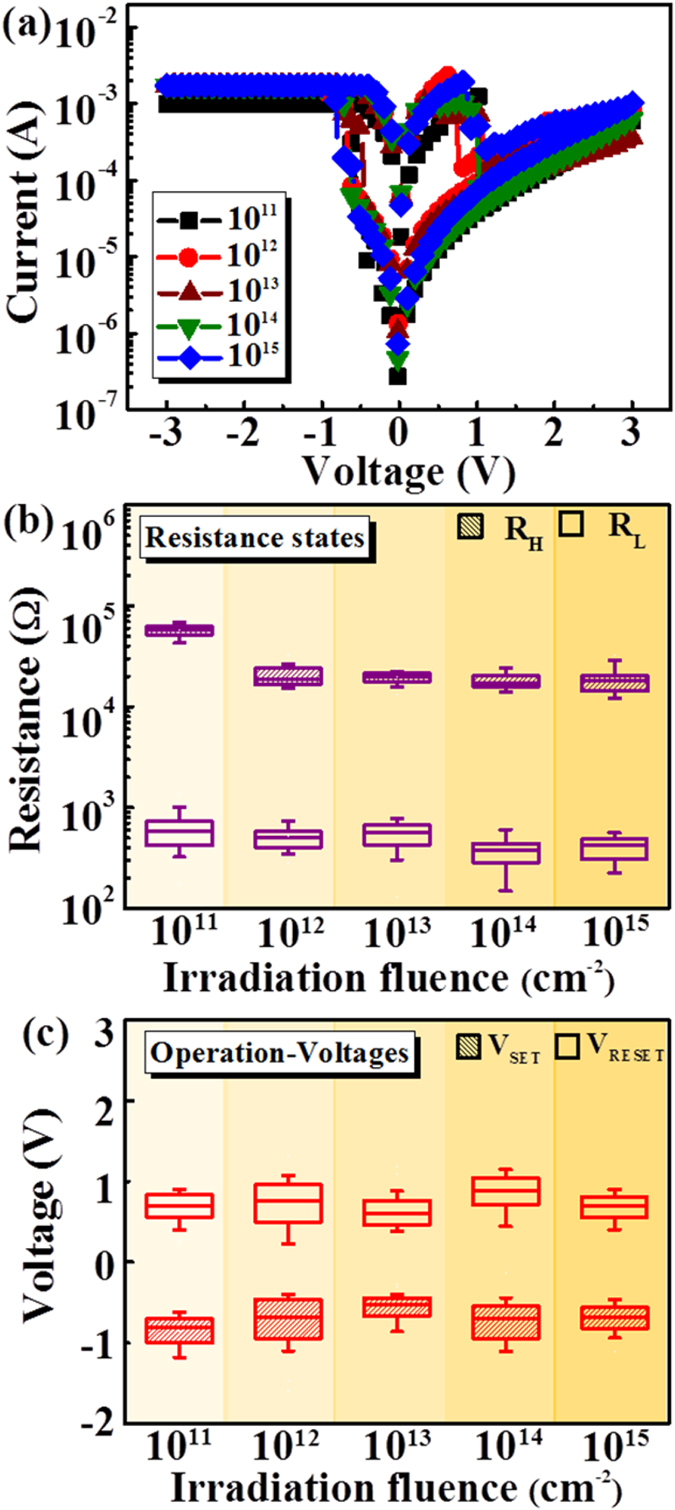
(**a**) *I*-*V* characteristics and the (**b**) resistance and (**c**) operation voltage distributions of the AlN TRRAM devices under proton irradiation fluences ranging from 10^11^ to 10^15^ cm^−2^.

To investigate the radiation tolerance of AlN TRRAM, we irradiated the device with 2 MeV proton fluences, ranging from 10^11^ to 10^15^ cm^−2^. It should be noted that protons with an energy less than 2 MeV and fluences ranging from 10^1^ to 10^8^ cm^−2^ occupy a significant volume of the Earth’s geomagnetic cavity (10–12 earth radii; *Re* = 6380 km)^4^ and thus can potentially affect electronic devices. The *I*-*V* characteristics of AlN TRRAM after different proton fluences are shown in Fig. 4a. We also performed a 100-cycle endurance test after each proton radiation exposure. Consequently, the distributions of the resistance states and operation voltages of the AlN TRRAM devices are shown in Fig. 4b and c, respectively. The results demonstrate narrow distributions and overall congruence. We also note that the resistance window (*R*_*H*_*/R*_*L*_) increased up to 30 regardless of proton irradiation fluences up to 10^15^ cm^−2^. Furthermore, these distributions were consistently similar to the control sample under air (Fig. 3a and b). It is also noteworthy that the transparency of the device did not degrade after proton irradiation^25^. Therefore, it is clear that the RS characteristics of the device are stable after proton irradiation of different fluences, suggesting a bright future for AlN-based TRRAM in aerospace applications.

## Conclusion

In this work, we investigated the reliability of AlN-based TRRAM for applications in harsh electronics. The AlN TRRAM exhibited an average transmittance of 80% in the visible wavelength range, and reliable resistive switching characteristics. Furthermore, statistical analyses of the cycle-to-cycle test in Fig. 2 and the device-to-device tests of 50 cells in Figs 3 and 4, demonstrated that AlN TRRAM is stable and can function properly under high oxygen partial pressure and proton irradiation. These experiments give insight not only into environmentally stable TRRAM design, but also for developing practical applications of TRRAM for future harsh electronics.

## Experimental Section

### TRRAM Fabrication

The structure of the AlN TRRAM device is shown in Fig. 1a. First, a 100 nm thick ITO thin film was deposited as the bottom electrode on a glass substrate by RF-sputtering (Ar flow 50 sccm, working pressure 3 m Torr, power 80 W). Second, a 50 nm thick AlN thin film was then deposited on the ITO layer by RF-sputtering (Ar flow 50 sccm, working pressure 5 mTorr. power 180 W). Finally, a patterned 100 nm thick ITO thin film was deposited as the top electrode by RF-sputtering (Ar flow 50 sccm, working pressure 3 mTorr, power 80 W) assisted with a metal shadow mask featuring an array of 200 μm diameter circles. These fabrication processes were carried out at room temperature.

### Characterization

The transmission spectrum of the fabricated device was measured by a JASCO V670 UV-VIS-NIR spectrophotometer. For radiation tolerance tests, each AlN TRRAM device was irradiated at room temperature using a 2 MeV proton beam produced from a 3 MV tandem accelerator (NEC 9SDH-2, National Electrostatics Corporation). The typical current of the proton beam was 2 nA to 50 nA with fluences ranging from 10^11^ cm^−2^ to 10^15^ cm^−2^ at the sample target. A Keithley 4200-SCS semiconductor characterization system was used to measure the resistive switching characteristics of the as-fabricated AlN TRRAM device.

## Additional Information

**How to cite this article:** Ho, C. H. *et al*. Transparent Memory For Harsh Electronics. *Sci. Rep.* **7**, 44429; doi: 10.1038/srep44429 (2017).

**Publisher's note:** Springer Nature remains neutral with regard to jurisdictional claims in published maps and institutional affiliations.
